# Severe Fever with Thrombocytopenia Syndrome Virus in Ticks and SFTS Incidence in Humans, South Korea

**DOI:** 10.3201/eid2609.200065

**Published:** 2020-09

**Authors:** Jeong Rae Yoo, Sang Taek Heo, Sung Wook Song, Seung Geon Bae, Seul Lee, Sungho Choi, Chaehyun Lee, Sugyeong Jeong, Myeongseop Kim, Woojin Sa, Yeongrim Lee, Haseon Choi, Sun-Ho Kee, Keun Hwa Lee

**Affiliations:** Jeju National University, Jeju, South Korea (J.R. Yoo, S.T. Heo, S.W. Song, S.G. Bae, S. Lee, S. Choi, C. Lee, S. Jeong, M. Kim, W. Sa, Y. Lee, H. Choi);; Korea University, Seoul, South Korea (S.-H. Kee);; Hanyang University College of Medicine, Seoul (K.H. Lee).

**Keywords:** Severe fever with thrombocytopenia syndrome virus, tickborne diseases, vector-borne infections, zoonoses, South Korea, viruses, severe fever with thrombocytopenia syndrome, SFTS, SFTSV

## Abstract

During 2016–2018, we collected 3,193 ticks from rural areas in South Korea to investigate the prevalence of severe fever with thrombocytopenia syndrome virus (SFTSV). We detected SFTSV in ticks at an infection rate (IR) of 11.1%. We noted increases in the human IR associated with the monthly SFTSV IR in ticks.

Severe fever with thrombocytopenia syndrome (SFTS) is a tickborne zoonosis caused by the SFTS virus (SFTSV) (*1*); >1,000 SFTS cases have been reported in South Korea (*2*). The SFTS prevalence rate was 2.26/100,000 inhabitants on the mainland and 13.66/100,000 inhabitants on Jeju Island, South Korea (*2*). SFTSV has been detected in several species of ticks, including *Haemaphysalis longicornis*, *Amblyomma testudinarium*, and *Ixodes nipponensis* (*3*). A previous study reported that the minimum infection rate of SFTSV in infected ticks was lower (0.37%) on Jeju Island than in other collection areas (1.97%) (*4*). However, 7%–14% SFTSV seropositivity was identified in domestic and wild animals (*4*–*5*), and 2%–5% SFTSV seropositivity was identified in a healthy population in South Korea (*6*). Therefore, our aim was to investigate the SFTSV infection rate (IR) in ticks in the region with the highest endemicity, Jeju Island, and to analyze the relationship between the geographic distribution of ticks and SFTSV and human cases of SFTS.

During June 2016–January 2019, well-equipped trained researchers collected ticks from the natural environment of Jeju Island. The tick sampling sites included 5 rural areas: Aewol-eup (AW); Seon Hul-ri (SH); Jeo Ji-ri (JJ); and Ha Do-ri (HD) and Bo Mok-ri (BM) (Figure). These 5 areas were chosen to compare SFTSV IR in ticks in areas with the highest rates of human SFTS cases, SH, HD, and AW, and SFTSV IR in ticks in areas with lower human SFTS rates, JJ and BM. Ticks were manually collected 2 times per month, during the first and third weeks, by dragging a white cloth in woodlands for 2 hours in each area. We morphologically identified tick species and developmental stages by using an Olympus SD-ILK-200–2 stereomicroscope (Olympus Corporation, https://www.olympus-lifescience.com) (*7*) and extracted viral RNA by using a QIAamp Viral RNA Mini kit (QIAGEN Inc., https://www.qiagen.com) according to the manufacturer’s instructions (Appendix).

**Figure:**
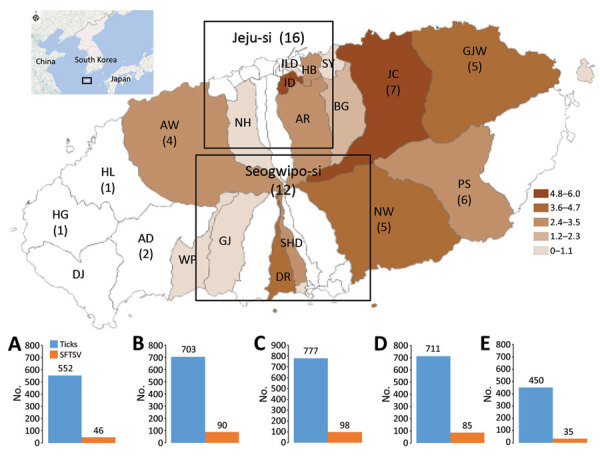
Geographic distribution of patients with severe fever with thrombocytopenia syndrome (SFTS) during January 2013–January 2019 and yearly incidence rates of SFTS virus (SFTSV) from June 2016–January 2019 on Jeju Island, South Korea. Inset shows location of Jeju Island near the coast of South Korea. Orange indicate regions of patients with SFTS in 2013–2019. Graphs show SFTSV detected in ticks and cases of human SFTS in A) Jeo Ji-ri; B) Aewol-eup; C) Seon Hul-ri; D) Ha Do-ri; and E) Bo Mok-ri. AR, Ara-dong; AW, Aewol-eup; BG, Bonggae-dong; BM, Bo Mok-ri; DR, Daeryun-dong; GJ, Ganjeong-dong; GJW, Gujwa-eup; HB, Hwabug-dong; HD, Ha Do-ri; ILD, Ildo-dong; ID, Ido-dong; JC, Jocheon-eup; JJ, Jeo Ji-ri; NH, Nohyeong-dong; NW, Nanwon-eup; PS, Pyoseon-myeon; SHO, Seohong-dong; SH, Seon Hul-ri; SY, Samyang-dong; WP, Wolpyeong-dong.

A total of 3,193 ticks were collected; most (99.9%) were *H. longicornis* and 81.3% of all ticks were nymphs. We detected SFTSV in 11.1% (354/3,193) of ticks (Appendix Table). Among the 5 areas, the average IR of SFTSV in ticks was 12.8% in AW, 12.6% in SH, 12.0% in HD, 8.3% in JJ, and 7.8% in BM. Adult ticks had a higher SFTSV IR (12.4%) than nymphs (9.5%). SFTSV was detected mainly in adult ticks (Appendix Table).

The monthly IR of SFTSV in ticks increased in May, peaked in July, and then slowly decreased (Appendix Figure 1). In addition, changes in the incidence of SFTS in patients were associated with increases in the monthly IR of SFTSV in ticks at a rate of 19.8% (95% CI 2.3%–40.2%) increase of SFTS in patients per 1% increase in monthly SFTSV IR in ticks (p = 0.02). SFTSV-infected ticks also were observed in the winter season. SFTSV sequences from infected ticks in our study and SFTS patients on Jeju Island were consistent with each other but differed from viruses in other regions of South Korea (Appendix Figure 2).

In a previous study, the prevalence of SFTSV in ticks was very low (0.2%), implying that ticks alone might not be sufficient to maintain SFTSV in nature (*8*). However, the high IR of SFTSV in ticks could explain why Jeju Island had one of the highest rates of human SFTS infection in South Korea. Changes in the incidence of patients with SFTS showed a pattern similar to that of monthly SFTSV IR in ticks. The IR of SFTSV in SH was the highest, along with HD and AW, where rates of human SFTS cases also were high. 

In East Asia, humans most frequently acquire SFTS during May–July, and shrub, forest, and rainfed cropland areas are associated with high risk for infection (*2*,*3*,*6*). Northeast Jeju Island, which includes SH, AW, and HD, has many farms and wetlands, and the IR of SFTSV in ticks peaked there in July, August, and September. In addition, SFTSV was detected in ticks in winter on Jeju Island, but no SFTS cases were reported in South Korea during winter. The 62 confirmed SFTS cases were statistically significantly associated with higher ambient temperature (22.5°C +4.2°C) compared with patients with negative RT-PCR results for SFTSV (18.9°C +5.7°C; p<0.001) (J.R. Yoo, unpub. data). The optimal temperature range for growth and reproduction of *H. longicornis* ticks is 20°C*–*24°C. Jeju Island maintains a temperature >20°C during May–October and is largely a rural and natural environment. We consider this area to have the highest prevalence of SFTS cases and ticks with SFTSV in South Korea. 

The results of this study showed that Jeju Island has the highest IR of SFTSV in ticks compared with other regions of South Korea and endemic countries. In addition, we found that the partial small segment of SFTSV in ticks was highly homologous to SFTSV in patients on Jeju Island and that Northeast Jeju Island, which includes SH, is a high-risk area for human SFTS infections.

This work was supported by a grant from the Korea Health Technology Research and Development Project through the Korea Health Industry Development Institute and funded by the Ministry of Health & Welfare, South Korea (grant no. HG18C0037).

Appendix Additional information on severe fever with thrombocytopenia syndrome (SFTS) virus in ticks and SFTS incidence in humans, South Korea.
