# Supplementary material for: Severe Fever with Thrombocytopenia Syndrome Virus in Ticks and SFTS Incidence in Humans, South Korea
Source: Emerg Infect Dis. 2020 Sep;26(9):2292–4. doi: 10.3201/eid2609.200065 (PMC7454071; doi:10.3201/eid2609.200065)
Supplement: Appendix — Additional information on severe fever with thrombocytopenia syndrome (SFTS) virus in ticks and SFTS incidence in humans, South Korea. [file 20-0065-Techapp-s1.pdf]

Article DOI: https://doi.org/10.3201/eid2609.200065

Severe Fever with Thrombocytopenia Syndrome Virus in Ticks and SFTS Incidence in Humans, South Korea

Appendix

Methods

Severe fever with thrombocytopenia syndrome viral RNA was extracted from ticks collected from Jeju Island, South Korea, by using a QIAamp Viral RNA Mini kit (QIAGEN Inc., https://www.qiagen.com) according to the manufacturer's instructions. Real-time reverse transcription PCR (RT-PCR) of the partial small (S) segment of SFTSV was performed for molecular diagnosis (*1*). The real-time RT-PCR products were sequenced by using a BigDye Terminator Cycle Sequencing kit (Perkin Elmer Applied Biosystems, www.perkin-elmer.com). Phylogenetic analyses of SFTSV partial S segment sequences from ticks and patients were conducted with MEGA6 (*2*), and phylogenetic trees were constructed using the maximum likelihood method.

Statistical analyses were performed by using SPSS 20.0 (IBM Corp., https://www.ibm.com) and $p<0.05$ was considered statistically significant. We used the annual number of cases of SFTS from the Disease Web Statistics System, Korea Centers for Disease Control and Prevention (http://is.cdc.go.kr). Binomial negative regression was used to analyze the monthly infection rates of ticks with SFTSV and SFTS patients on Jeju Island.

References

1. Yun Y, Heo ST, Kim G, Hewson R, Kim H, Park D, et al. Phylogenetic analysis of severe fever with thrombocytopenia syndrome virus in South Korea and migratory bird routes between China, South Korea, and Japan. Am J Trop Med Hyg. 2015;93:468–74. PubMed https://doi.org/10.4269/ajtmh.15-0047

2. Tamura K, Stecher G, Peterson D, Filipski A, Kumar S. MEGA6: Molecular Evolutionary Genetics Analysis version 6.0. Mol Biol Evol. 2013;30:2725–9. PubMed https://doi.org/10.1093/molbev/mst197

Appendix Table. Severe fever with thrombocytopenia syndrome virus detection in 3,193 ticks by developmental stage and region, Jeju Island, South Korea*

	JJ		AW		HD		BD		SH		
Stage	No.	SFTSV (IR, %)	No.	SFTSV (IR, %)	No.	SFTSV (IR, %)	No.	SFTSV (IR, %)	No.	SFTSV (IR, %)	Total, %
Adult, F	51	3 (5.9)	73	13(17.8)	76	15 (19.7)	55	4 (7.3)	98	15 (15.3)	12.7
Adult, M	25	3 (12.0)	52	7 (13.5)	52	9 (17.3)	32	0	80	13 (16.3)	12.0
Nymph	476	40 (8.4)	578	70 (12.1)	583	61 (10.5)	362	31 (8.6)	598	70 (11.7)	9.5
Larva	0	0	0	0	0	0	1	0	0	0	0
Total	552	46 (8.3)	703	90 (12.8)	711	85 (12.0)	450	35 (7.8)	777	98 (12.6)	11.1

*AW, Aewol-eup; BM, Bo Mok-ri; Ha Do-ri; HD, IR, infection rate of SFTSV in ticks; JJ, Jeo Ji-ri; SH, Seon Hul-ri; SFTSV, severe fever with thrombocytopenia syndrome virus.

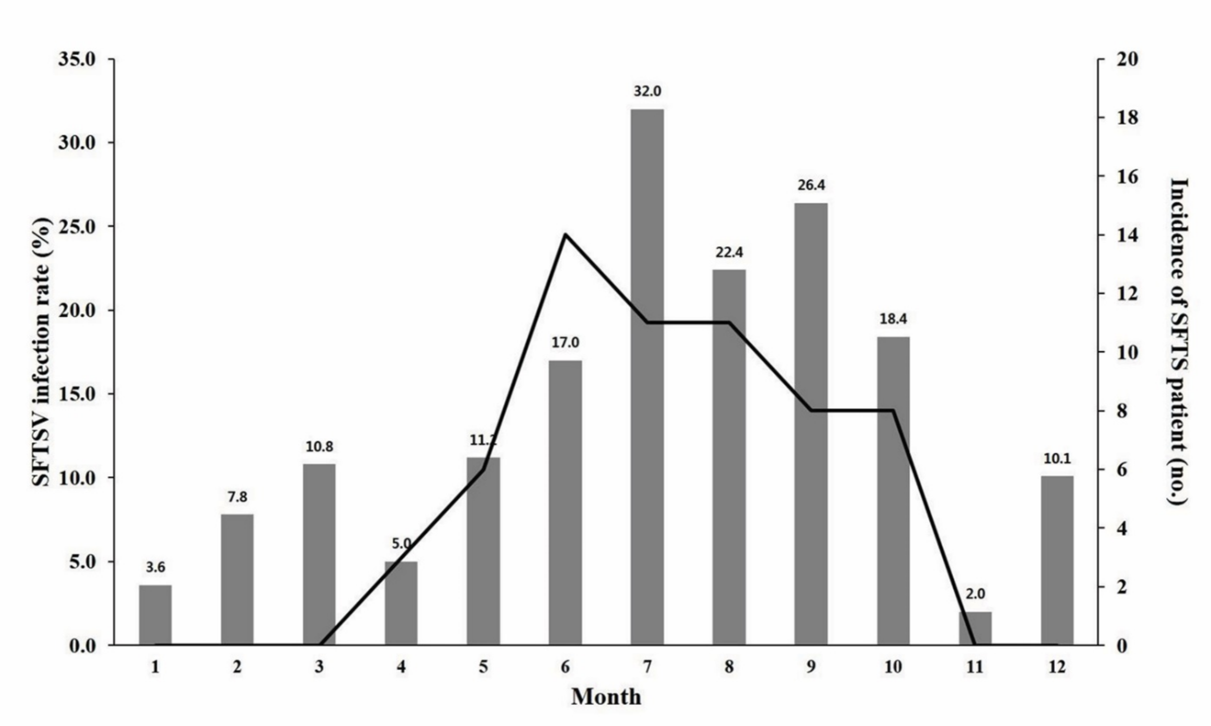

Appendix Figure 1. Monthly severe fever with thrombocytopenia syndrome virus infection rate in ticks (2016–2018) and incidence of patients with SFTS (2013–2019) on Jeju Island, South Korea. Black line indicates the monthly incidence of SFTS in patients on Jeju Island (2013–2019). Gray bars indicate the monthly SFTSV infection rate in ticks on Jeju Island (2016–2018). SFTS, severe fever with thrombocytopenia syndrome; SFTSV, severe fever with thrombocytopenia syndrome virus.

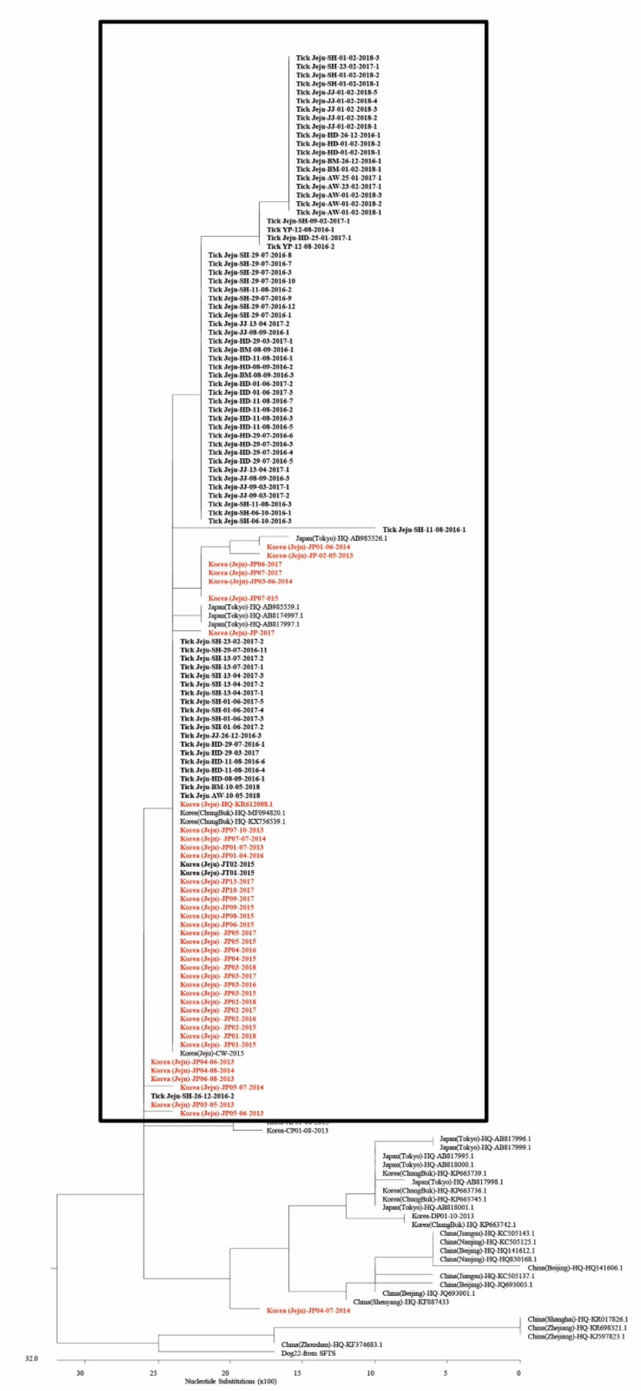

Appendix Figure 2. Phylogenetic tree based on partial small (S) segment sequences severe fever with thrombocytopenia syndrome (SFTS) virus. The tree was constructed by using the maximum likelihood method in MEGA 6 (*2*). The partial S sequences were obtained from ticks during June 2016–January 2019. The partial S sequence data for the viruses identified in China, South Korea, and Japan were obtained from NCBI/BLAST (https://blast.ncbi.nlm.nih.gov/Blast.cgi).
